# Difference in C_3_–C_4_ metabolism underlies tradeoff between growth rate and biomass yield in *Methylobacterium extorquens* AM1

**DOI:** 10.1186/s12866-016-0778-4

**Published:** 2016-07-19

**Authors:** Yanfen Fu, David A. C. Beck, Mary E. Lidstrom

**Affiliations:** Department of Chemical Engineering, University of Washington, 616 NE Northlake Place, Benjamin Hall Room 440, Seattle, 98105 WA USA; Department of Microbiology, University of Washington, 616 NE, Northlake Place, Seattle, 98195 WA USA; eScience Institute, University of Washington, 616 NE, Northlake Place, Seattle, 98195 WA USA

**Keywords:** *Methylobacterium extorquens* AM1, Methylotrophy, Physiological trade-off, Metabolic flux analysis, Cobalt

## Abstract

**Background:**

Two variants of *Methylobacterium extorquens* AM1 demonstrated a trade-off between growth rate and biomass yield. In addition, growth rate and biomass yield were also affected by supplementation of growth medium with different amounts of cobalt. The metabolism changes relating to these growth phenomena as well as the trade-off were investigated in this study. ^13^C metabolic flux analysis was used to generate a detailed central carbon metabolic flux map with both absolute and normalized flux values.

**Results:**

The major differences between the two variants occurred at the formate node as well as within C3–C4 inter-conversion pathways. Higher relative fluxes through formyltetrahydrofolate ligase, phosphoenolpyruvate carboxylase, and malic enzyme led to higher biomass yield, while higher relative fluxes through pyruvate kinase and pyruvate dehydrogenase led to higher growth rate. These results were then tested by phenotypic studies on three mutants (null *pyk*, null *pck* mutant and null *dme* mutant) in both variants, which agreed with the model prediction.

**Conclusions:**

In this study, ^13^C metabolic flux analysis for two strain variants of *M. extorquens* AM1 successfully identified metabolic pathways contributing to the trade-off between cell growth and biomass yield. Phenotypic analysis of mutants deficient in corresponding genes supported the conclusion that C3–C4 inter-conversion strategies were the major response to the trade-off.

**Electronic supplementary materials:**

The online version of this article (doi:10.1186/s12866-016-0778-4) contains supplementary material, which is available to authorized users.

## Background

**Fig. 1:**
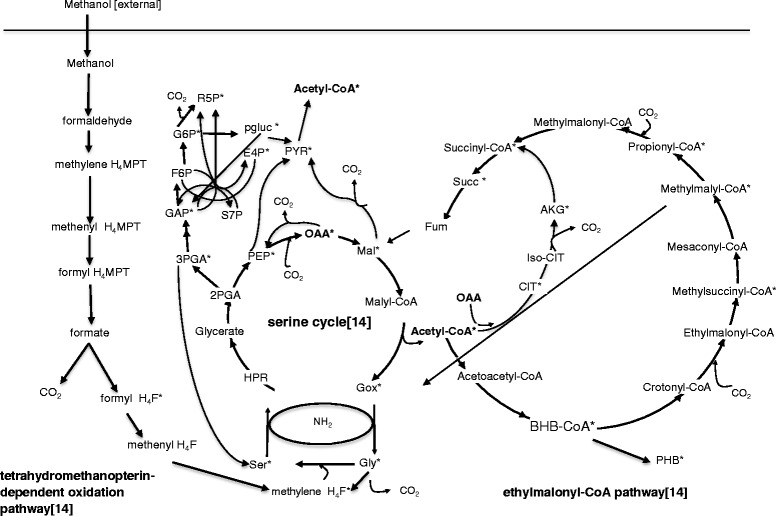
Central carbon metabolism model for *M. extorquens* AM1 methylotrophic growth. Metabolites with * are precursors for biomass. Metabolites in bold are branch points. The model includes 114 reactions with 9 reversible reactions and 2 scramble reactions. 71 intracellular metabolites are included in the model. Methanol is oxidized to formate via the H_4_MPT pathway. Part of the formate pool is converted to CO_2_ by formate dehydrogenase. The other part is converted into methylene H_4_F via the H_4_F pathway, entering the serine cycle. The serine cycle is the main assimilation pathway, with the EMC pathway regenerating glyoxylate in an anaplerotic function. Portions of the TCA cycle, gluconeogenesis, and the pentose-phosphate cycle were also operating to provide intracellular metabolites for biomass

*Methylobacterium extorquens* AM1 is a facultative α-proteobacterial methylotroph, that has been studied intensively over 50 years [1]. The availability of the genome sequence for *M. extorquens* AM1 [2]. intensive developments of genetic tools [3, 4], and well-studied biochemistry and physiology have made the organism a model system for C1 metabolism. With the development of transcriptomics, proteomics, metabolomics and fluxomics, studies on C1 metabolism in *M. extorquens* AM1 have recently been carried out using system approaches [5–7]. C1 metabolism involves multiple C1-specific metabolic pathways, including the tetrahydromethanopterin-dependent oxidation pathway, the serine cycle, and the ethylmalonyl-CoA pathway as shown in Fig. 1.

Recent studies have shown that cobalt is an important trace metal for methylotrophic growth in *M. extorquens* AM1. Cobalt is needed for vitamin B_12_ production used as cofactor for two enzymes involved in methylotrophy growth, methylmalonyl-CoA mutase (Mcm) and ethylmalonyl-CoA mutase (Ecm) in the ethylmalonyl-CoA pathway, and plays a role in strain fitness [8, 9, 11]. Three research groups have published optimized media recipes, including optimization of cobalt levels [8–11]. However, the effect of cobalt on the overall central carbon metabolism in *M. extorquens* AM1 remains unknown.

Strain integrity can become compromised when the same strain is transferred between labs using different storage procedures, as illustrated recently for *M. extorquens* AM1 [12]. Phenotypic divergence was observed between an archival strain and a modern strain in terms of growth rate and fitness across various culture conditions [12]. The literature shows that two other strains have diverged in Mary Lidstrom’s lab and Julia Vorholt’s lab, after these strains were separated for 14 years. Different growth rates were reported from previous studies for both strains [13, 14], which could be ascribed to a combination of culturing environment and unintended domestication of the *M. extorquens* AM1 strain, but the basis for this difference is not known.

It has been well-documented that a trade-off exists between rate and yield for heterotrophic organisms in which growth rate is predicted to be limited by ATP [15, 16]. However, it was not known whether such a tradeoff occurs in the *M. extorquens* AM1 strain variants. In *M. extorquens* AM1, the cell growth is predicted to be limited by reducing power instead of ATP [13], making the metabolic basis for such tradeoffs unclear. The availability of two strain variants with differences in growth rate and possibly in biomass yield offers an opportunity to decipher system-wide metabolic responses in *M. extorquens* AM1, including the possible trade-off between growth rate and biomass yield.

^13^C metabolic flux analysis is a powerful tool, which combines both experimental and computational approaches to quantitatively understand the metabolic pathways in a living organism. It is based on a stoichiometric reaction model and extracellular consumption and secretion, along with ^13^C labeling information to calculate in vivo reaction rates [17–21]. It generates both flux maps with absolute values as well as flux distribution normalized to specific substrate uptake rate, offering insights into cell behavior at the metabolic activity level. This technique provides an approach to address the metabolic changes underlying the physiological differences observed in the two *M. extorquens* strain variants noted above.

In this study, it was established that a tradeoff exists between growth rate and biomass yield in these two *M. extorquens* AM1 strain variants, and ^13^C metabolic flux analysis (MFA) was applied to both strain variants in order to assess the metabolic differences underlying the trade-off. In addition, MFA was applied to the same strain with different cobalt supplements to assess the effect of cobalt on overall central carbon metabolism and its contribution to the trade-off.

## Results

### Tradeoff between growth rate and biomass yield exists in *M. Extorquens* AM1 for two strain variants as well as two cobalt levels

Observations of growth rate differences prompted a more in-depth study of two *M. extorquens* AM1 strains, a parent strain from the laboratory of Mary Lidstrom (LL strain), and a strain that originated from the Lidstrom laboratory, but was carried in the laboratory of Julia Vorholt (VL strain) since 2001. Both genomes of the two variants were sequenced in this study. A small number of genomic differences were detected between three different *M. extorquens* strains (VL, LL and Chris Marx modern strain [22]) (see Additional file 1: Table S1). However, these differences did not provide an obvious solution to the trade-off or suggest how the divergence in genome sequence affects growth rate. Growth experiments were conducted for the two strains with methanol as the sole carbon substrate. A minimal medium from the Lidstrom laboratory (called hypho medium, HY) was used to be consistent with previous work done in the Lidstrom laboratory. In addition, since levels of cobalt in the medium also affect growth rate of *M. extorquens* AM1 strains [8, 9] but the effect on biomass yield is unknown, this factor was also studied. Three different levels of cobalt were tested for growth rate experiments (data not shown). For both strains, the most dramatic differences were between 1.35 and 6.31 μM. These two concentrations were then chosen to test the effect of cobalt supplementation.

**Fig. 2:**
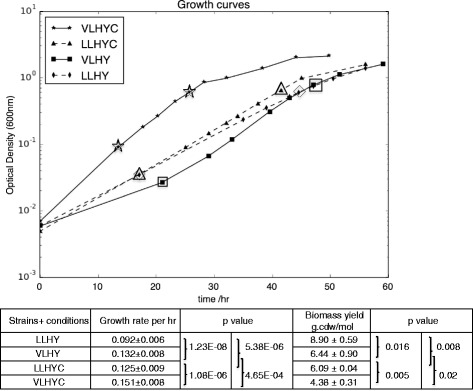
Representative growth curves for two strain variants with different concentration of cobalt supplement. Time points for supernatant methanol concentration measurements were highlighted by shapes corresponding to each condition. Growth rate and biomass yield were reported for each condition with *p* values from student *t*-test with *n* > =3

Biomass yield on methanol, which was defined as g cell dry weight generated per mol methanol consumption, was monitored by measuring methanol concentration in supernatant and biomass concentration during log phase (at least two time points were taken during log phase). Excretion products were tested in culture supernatant and were found to be negligible. A negative correlation between growth rate and biomass yield was observed, with both strain difference and cobalt level playing a role in the trade-off. As shown in Fig. 2, the VL strain grew 22 % faster than the LL strain in the presence of 1.35 μM cobalt (normal HY medium), and similarly, 21 % faster in medium with 6.31 μM cobalt (termed HYC medium). However, biomass yield shows the opposite trend. The biomass yield of was 28 % lower than that of the LL strain in both HY and HYC. A similar trade-off was also observed for the same strain growing in medium with different cobalt levels. The LL strain grew 26 % faster but with 32 % lower biomass yield in HYC medium than in HY medium, while the VL strain grew 25 % faster with 28 % lower biomass yield in HYC than HY.

In summary, within the four conditions tested in this study, LL in HY medium has the slowest growth rate but highest biomass yield while VL in HYC medium has the fastest growth rate but lowest biomass yield. Since both strain difference and cobalt level were taken into account in this set of comparisons, analysis of the metabolic response to the trade-off includes both factors.

### Flux distribution reveals different metabolic strategies in C_3_–C_4_ interconversions and the formate branch node

**Fig. 3:**
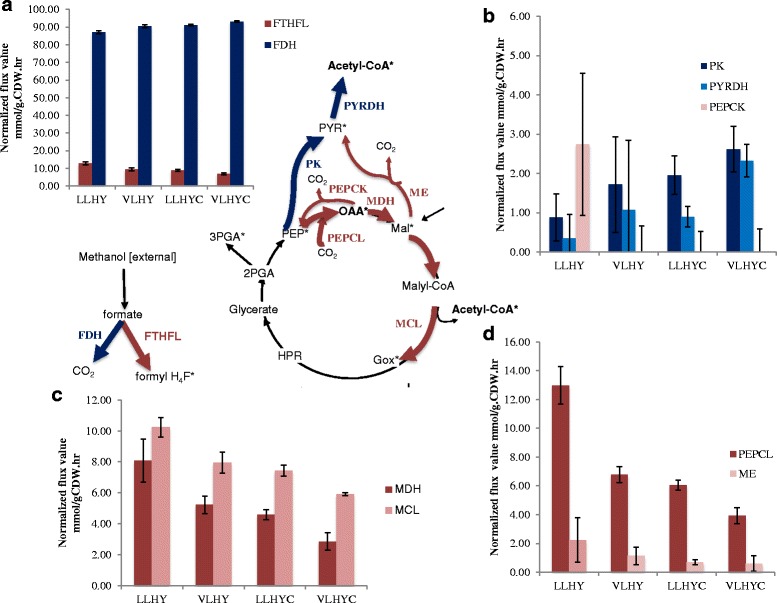
Flux distribution map showing flux values normalized to the methanol uptake rate. The major differences are highlighted in colors. In green, the increased fluxes correlated with higher growth rate; in red and pink, the increased fluxes correlated with higher biomass yield. **a** flux partition at the formate node; **b** fluxes from PEP to AcCoA using PK and PYRDH pathway, and OAA to PEP with PEPCK. **c** flux through MDH and MCL. **d** flux through PEPCL and ME. Data represent the averages from 3 biological replicates ± standard deviations

In order to assess metabolic differences between the two strains, detailed central carbon flux maps were generated for each strain to provide a quantitative description for the two growth conditions using ^13^C metabolic flux analysis (^13^C-MFA). The published flux balance model and biomass composition for the VL strain [13] was used as a basis for a model developed in Influx_s. The specific methanol uptake rate of cells was measured in exponential phase, and an analysis of extracellular metabolites for cells grown in the same way showed negligible amounts. The model consists of 114 reactions including 9 reversible reactions and 2 scrambling reactions. A total of 149 MS measurements were used for model fitting with 12 calculated label measurements. The simulated flux data were then normalized to a specific methanol uptake rate to generate the flux distribution map for each condition. The flux distribution comparison shown in Fig. 3 indicates the major differences were in the formate branch point and in C3-C4 interconversion pathways. As shown in the central carbon metabolism map, formate could either be oxidized to CO_2_ by formate dehydrogenase (FDH) or be converted into formyl-H_4_F by formyltetrahydrofolate ligase (FTHFL). As expected by the growth rate difference, the absolute methanol uptake rate and flux through formate to both branches was higher for the VL strain than the LL strain (see Additional files 2, 3, 4 and 5: Table S2, S3, S4 and S5). However, the flux distribution across the branch point, which is normalized to the methanol uptake rate, shows a strain-specific difference in keeping with the higher biomass yield for the LL strain. For the LL strain, FTHFL carries 36 % more flux entering the assimilation pathway than the VL strain in HY, 28 % more in HYC.

Likewise, two routes exist for conversion of phosphoenolpyruvate (PEP) into acetyl-CoA (AcCoA). In the first route, the classic serine cycle involves conversion of PEP into oxaloacetate (OAA) through a carboxylation pathway involving PEP carboxylase (PEPCL), and then conversion of OAA into malate (MAL) by malate dehydrogenase (MDH), which is then further converted into glyoxylate and AcCoA by malyl-coA lyase (MCL). A reaction also exists to decarboxylate OAA back to PEP by phosphoenolpyruvate carboxykinase (PEPCK). An alternative route utilizes PEP to synthesize pyruvate through pyruvate kinase (PK), and then pyruvate is decarboxylated by pyruvate dehydrogenase (PYRDH) to produce AcCoA. Pyruvate could also be produced from decarboxylation of malate through malic enzyme (ME). The flux distribution shows that PEPCL had a higher normalized flux in the LL strain than the VL strain by 91 % in HY and by 55 % in HYC. For each strain grown at different cobalt concentrations, the normalized PEPCL flux decreased by 53 % at the higher cobalt concentration in HYC for the LL strain and 42 % for the VL strain. PEPCK is active for the LL strain in HY, but is negligible for the LL strain in HYC. For the VL strain, PEPCK is negligible regardless of the level of cobalt. For the malic enzyme pathway (ME), the LL strain has 97 % higher flux than the VL strain in HY, but the difference became smaller in HYC, with the higher cobalt concentration. PK in the LL strain is 49 % lower than VL in HY with the lower cobalt level, 25 % in HYC. A similar trend was observed for PYRDH for both conditions but to different extents, 67 and 61 % respectively.

### Phenotypic differences of mutants

Based on the flux analysis results, three genes were targeted for mutation (*dme* encoding ME, *pck* encoding PEPCK and *pyk* encoding PK), to assess mutant phenotypes in the two strains. It is known that PEPCL, MCL, and MDH are required for growth on methanol [23] so those mutants were not generated.

From the metabolic flux distribution result, the following phenotypes were predicted. The growth rate was associated with the PYK pathway encoded by *pyk*. Conditions with faster growth have higher flux through PYK. The *pyk* mutant should have higher negative impact on growth rate for the VL strain under both medium conditions, while for the LL strain the impact should be minor. The growth rate experiments on *pyk* mutants agree with this prediction. As shown in Table 1, for HY medium, LL: Δ*pyk* shows similar growth as LL, while VL: Δ*pyk* has 56 % slower growth rate than VL. For HYC medium, LL: Δ*pyk* shows a 13 % growth defect compared to LL under the same condition, while VL: Δ*pyk* has a 36 % slower growth rate than VL under the same condition. The metabolic flux distributions can only predict the impact of this mutation on growth rate, but it is also of interest to assess impact on yield. The biomass yield of LL: Δ*pyk* and VL: Δ*pyk* showed minor changes in HY medium, while in HYC medium, biomass yield increased slightly for both strains. This suggests that in the Δ*pyk* mutant, the route of the standard serine cycle with PEPCL, MDH and ME carries more flux than in the original strain, which can lead to higher biomass yield.

**Table 1:** Growth rate and biomass yield for *dme*, *pck*, and *pyk* mutants of both strain variants, base cases included, marked as^a^

Strain + medium condition	Biomass yield (g cdw/mol methanol consumed)	Absolute change in biomass yield (g cdw/mol methanol consumed)	Percent of original strain biomass yield under same condition	Growth rate (per hr)	Absolute change in growth rate(per hr)	Percent of original strain growth under same condition
LL + HY	8.90 ± 0.59	^a^	^a^	0.09 ± 0.01	^a^	^a^
VL + HY	6.44 ± 0.90	^a^	^a^	0.13 ± 0.01	^a^	^a^
LL + HYC	6.09 ± 0.04	^a^	^a^	0.13 ± 0.01	^a^	^a^
VL + HYC	4.38 ± 0.31	^a^	^a^	0.15 ± 0.01	^a^	^a^
LL: D*pck* + HY	4.84 ± 0.98	−4.07	54 % ± 11 %	0.10 ± 0.01	0.01	111 % ± 12 %
VL: D*pck* + HY	5.26 ± 0.52	−1.17	82 % ± 8 %	0.10 ± 0.01	−0.03	77 % ± 8 %
LL: D*pck* + HYC	4.76 ± 1.67	−1.33	78 % ± 27 %	0.09 ± 0.01	−0.04	75 % ± 10 %
VL: D*pck* + HYC	5.06 ± 0.49	0.68	115 % ± 11 %	0.09 ± 0.01	−0.06	60 % ± 8 %
LL: D*dme* + HY	4.07 ± 2.06	−4.83	46 % ± 23 %	0.09 ± 0.01	0	100 % ± 10 %
VL: D*dme* + HY	4.62 ± 1.02	−1.81	72 % ± 16 %	0.09 ± 0.01	−0.04	69 % ± 8 %
LL: D*dme* + HYC	5.92 ± 0.37	−0.16	97 % ± 6 %	0.07 ± 0.01	−0.06	58 % ± 10 %
VL: D*dme* + HYC	6.01 ± 0.76	1.63	137 % ± 17 %	0.10 ± 0.01	−0.05	67 % ± 7 %
LL: D*pyk* + HY	7.77 ± 0.45	−1.13	87 % ± 6 %	0.10 ± 0.01	0.01	108 % ± 6 %
VL: D*pyk* + HY	6.30 ± 0.17	−0.14	98 % ± 4 %	0.06 ± 0.01	−0.07	44.9 % ± 6 %
LL: D*pyk* + HYC	7.60 ± 0.56	1.51	125 % ± 8 %	0.11 ± 0.01	−0.02	86 % ± 10 %
VL: D*pyk* + HYC	6.63 ± 1.14	2.25	151 % ± 17 %	0.10 ± 0.01	−0.05	63 % ± 10 %

**Table 2:** Strains and plasmid

Strain	Plasmid/Genotype	Reference
LL	Rif derivative	M. Lidstrom
VL	Rif derivative	J. Vorholt
FYF1	LL:: Δ*dme*	This study
FYF2	VL: Δ*dme*	This study
FYF3	LL:: Δ*pck*	This study
FYF4	VL:: Δ*pck*	This study
Plasmids	Description	Reference
pCM184	Ap^r^, Kn^r^,Tc^r^; pCM182 with *kan* from pCM183; allelic exchange vector	3

Secondly, metabolic flux map results also indicate that the non-essential pathways associated with biomass yield were ME encoded by *dme* and PEPCK encoded by *pck*. The higher biomass yield conditions have higher carbon flux through both ME and PEPCK. This predicts that both of these mutants should have a more negative impact on conditions with higher biomass yield. The experimental results on biomass yield for these two mutants agree with the prediction shown in Table 1. As shown in Table 1, the *dme* mutation had a negative effect on biomass yield in the LL strain in HY medium (54 % decrease) compared to wild type LL. A smaller decrease in biomass yield (28 %) occurred in VL: Δ*dme*. Likewise, the *pck* mutation also had a negative impact on biomass yield in the LL strain (46 % decrease compared to wild type LL), while for VL: Δ*pck* in HY, it decreased by only 18 %. The effect on biomass yield for both strains was less when the cells were grown with the higher level of cobalt. For both VL: Δ*dme* and VL: Δ*pck*, the biomass yield increased. Noticeably, the *dme* mutant in VL and LL strain had the same biomass yield in HYC when extra cobalt was supplied to the medium, while the difference was more pronounced in HY medium. The Δ*pck* and Δ*dme* strains did not have large impacts on growth rate, but showed different extents of growth defects (Table 1). These two pathways involve NADH and ATP consumption, respectively. Therefore these two mutants are expected to alter consumption of ATP and NADH, which might be expected to affect growth rate.

## Discussion

This work generated four detailed flux distribution maps for two strain variants of *M. extorquens* AM1 (the LL and VL strains) grown with different cobalt levels. ^13^C MFA has previously been applied to the VL strain during growth on methanol at a higher cobalt level [13]. However, it is not possible to compare the flux maps directly, as in the previous study the culture was grown with 5 % CO_2_ purging the culture system. The influence of 5 % CO_2_ on metabolism in *M. extorquens* AM1 is unknown, but given the number of carboxylation and decarboxylation reactions involved in growth on one-carbon compounds, it might alter fluxes. In this study we used air (0.5 % CO_2_) and also a slightly different medium recipe, to allow direct comparisons with previous metabolic studies of this bacterium carried out in this medium with 0.5 % CO_2_.

We have shown that the VL strain grew faster with lower biomass yield than the LL strain, regardless of the cobalt supplementation. From these results we conclude that a tradeoff exists between growth rate and biomass yield that is strain-specific. This suggests that these two strains are an example of unintended domestication, which has been reported previously for *M. extorquens* AM1 [12]. In addition, the cobalt level also affects growth rate and biomass yield in a manner that is not strain-specific. Improved growth rate and increased absolute fluxes through the EMC cycle in response to higher cobalt are in keeping with previous studies [8, 11] suggesting that the EMC pathway could be a potential bottleneck for methanol assimilation under cobalt limitation conditions.

The occurrence of natural strain variants with growth rate/biomass yield tradeoffs along with the cobalt affect provided the opportunity to determine features of metabolic network response underlying these major physiological attributes of growth rate and biomass yield. ^13^C-flux labeling revealed that FTHFL, the pathway involving the standard serine cycle (PEPCL, MDH, and MCL), and PEPCK and ME all had higher relative flux in conditions when biomass yield was higher, while the alternate pathway to AcCoA involving PK and PYRDH had higher relative flux in conditions when growth rate was faster. These differences were predicted to affect NADH and ATP usage. PEPCK consumes one ATP, while PK and PYRDH produce one ATP and one NADH, respectively. MDH and MCL consume NADH and ATP, respectively. These results suggest that higher biomass yield is achieved under conditions that result in relatively higher relative fluxes through the standard serine cycle-based carbon assimilation pathways, even though this metabolic scheme consumes more NADH and ATP than the one involving conversion of pyruvate to AcCoA, at the expense of growth rate. Likewise, higher growth rate is achieved by diverting carbon through pathways with higher NADH and ATP production, with concomitant decrease in biomass yield.

There must be a genetic explanation for the trade-off. However, genome sequence results did not provide a clear clue, since none of the genomic changes is predicted to directly impact any of the C3/C4 reactions that show altered flux. One of the possible explanations is that the mutants in the gene cluster with unknown functions lead to shifted flux through C3/C4 enzymes due to the change in small molecule pools, which in turn affects yield versus growth rate.

The results of mutant phenotypes are consistent with these conclusions regarding metabolic network response, since loss of the biomass yield-related enzymes resulted in an impact on biomass yield, and loss of a growth-rate related enzyme resulted in an impact on growth rate. It is more difficult to predict the impact of the *pyk* mutant on yield, and of the *dme* and *pck* mutants on growth rate. C3-C4 interconversion pathways in this bacterium are flexible due to the fact that some of the enzymes are reversible (such as MDH, ME) as well as the fact that different enzymes are present that catalyze forward and reverse reactions (such as PK, PEP synthase, PEPCL and PEPCK). In the two strain variants, two C3/C4 pathways dominate but with different ratios, as shown in Fig. 3, but we do not know how the network will change in the mutants. Therefore, although we can predict that removing the optimal variant would decrease growth rate or yield, respectively, the mutation may or may not also affect the other parameter, depending on how the metabolic network rearranges.

The cobalt concentration used in the growth medium also affected the fluxes and mutant phenotypes. In both strains, growth in higher cobalt resulted in higher growth rates and a correspondingly higher absolute flux through the assimilatory pathways. Since the EMC pathway contains two steps requiring B_12_-utilizing mutases (ethylmalonyl-CoA mutase and methylmalonyl-CoA mutase), it is expected that higher availability of cobalt would affect flux through the EMC shown by others [8, 11]. The increased absolute flux through the EMC suggests that a cobalt-related component of one or both of these enzymes might limit flux under the lower cobalt growth condition. However, although the absolute flux increased through all of the central assimilatory pathways with increased cobalt, the flux distribution (normalized to the methanol uptake rate) did not. The flux distribution through the EMC was essentially the same for both the high and low cobalt conditions. This result suggests that the assimilatory flux is well balanced with both the methanol uptake rate and the formate branchpoint distribution, and that the metabolic network has the capacity to increase flux to this level. The significant changes in the flux distribution that occur in response to cobalt are not in the EMC pathway, but instead, are in the nexus between the serine cycle, the partial TCA cycle, the pentose-phosphate pathway, and the EMC pathway. It seems likely that this redundancy in C3–C4 interconversions at this metabolic intersection provides robustness for the metabolic network. The mechanism(s) for these changes are not yet known, but could occur at the transcriptional or post-transcriptional level.

The flux distribution maps presented here provide information that is potentially useful in guiding strain development for biotechnical applications. For applications in which faster strain growth is favored to create optimal economic conditions for a product (for instance, when a biomass product is the target), reactions identified in this study associated with faster growth are targets for manipulation. Similarly, if instead higher biomass yield is favored, for instance with an excreted product, the reactions associated with higher biomass yield could be manipulation targets. It should also be noted that as shown in this study, a single trace mineral change in the medium changes the flux distribution, pointing out the importance of flux analysis in assessing metabolic dynamics and growth conditions.

## Conclusion

Growth rate and biomass yield measurements for two culture conditions with two strain variants suggest trade-offs exist between growth rate and biomass yield. ^13^C flux analysis was successfully applied to identify metabolic differences that could contribute to the trade-off. Pathways with different activities were identified, which were used to generate a hypothesis of the metabolic changes responding to the trade-off, focused on C3–C4 inter-conversion strategies. The hypothesis was tested using mutants, and their phenotypes supported the conclusion that the flux difference mainly involves C3–C4 interconversion strategies.

## Methods

### Chemicals and medium composition

^13^C methanol of 99 % purity was purchased from Cambridge Isotope Laboratories (Tewksbury, MA). All other chemicals including metabolite standards were purchased from Sigma-Aldrich (St. Louis, MO). Phusion DNA polymerase, dNTP, buffer, ligases, OneTaq Quick-Load 2X Master Mix and the Gibson Assembly Master Mix kits used in this study were from New England Biolabs (Ipswich, MA). Primers, the sequences of which are shown in Table 1, were obtained from Invitrogen (Grand Island, NY) and IDT (Coralville, IA). Acetonitrile and water used as UPLC solvents were UPLC-MS grade. Hypho minimal medium as previously described [24] was used for ^13^C flux analysis cell culture and growth rate determination, involving two different concentrations of cobalt (1.35 and 6.31 μM respectively).

### Bacterial strains, plasmid and growth condition

Strains used in this study are listed in Table 2. *Escherichia coli* strains Top 10 and S17-1 were cultivated at 37 °C in Luria-Bertani medium. Two wild type *M. extorquens* AM1 strain were used, in which VL is from the Julia Vorholt lab and LL is from the Mary Lidstrom lab. Plasmid pCM184 was used as template for backbone and *cre*-*kan* amplification. A Gibson assembly kit (New England Biolabs, Ipswich, MA) was used for plasmid construction to create knock out strains with a kanamycin marker FYF1, FYF2, FYF3 and FYF4, (primer sequences shown in Table 3). Insertion mutants of *pyk* were generated for both LL and VL variants as described earlier [25] because of our inability to obtain clean knockout using the method described in this study. 125 mM methanol was used as the sole carbon source for cell culture and growth curves. For ^13^C flux analysis, ^13^C methanol was introduced at the stage of 3 ml seed culture in tubes, and then inoculated into flasks with screw caps in a dilution ratio of 1:200 in triplicates. Cell culture was then quenched using fast filtration and liquid nitrogen after the cell reaches OD_600_ around 0.6–0.8 (7 generations). For normal growth curve cell culture, methanol was added as carbon source in 3 ml seed culture, and then inoculated into flasks with screw caps with the same dilution ratio.

**Table 3:** Gene sequences for primers used for PCR amplification in this study

Primer name	Primer description	Sequence
YFP1	dme upstream flank forward	cgcacatttccccgaaaagtgccacctgacgtctagatctatccaccgcatcaccgtctc
YFP2	dme upstream flank backward	tggcataacttcgtataatgtatgctatacgaagttatgggcactgttgaggcatctgtt
YFP3	dme downstream flank forward	ctcagcgataacttcgtatagcatacattatacgaagttagcattcagttccgggcgttg
YFP4	dme downstream flank backward	gcgtggaaagcctggtcggctggatcctctagtgagctcgcccttcttctccttggtcca
YFP5	pck upstream flank forward	cgcacatttccccgaaaagtgccacctgacgtctagatctgatcggacacgtcctccacc
YFP6	pck upstream flank backward	acgtggcataacttcgtataatgtatgctatacgaagttatggggtgttcctccctcacg
YFP7	pck downstream flank forward	gacctcagcgataacttcgtatagcatacattatacgaagttagcggtcacagcccctcc
YFP8	pck downstream flank backward	cgtggaaagcctggtcggctggatcctctagtgagctccgagccgctctacaatctcgac
YFP9	pCM184 cre-km forward	ataacttcgtatagcatacattatacgaagttatgccacgttgtgtctcaaaatctctg
YFP10	pCM184 km-cre backward	taacttcgtataatgtatgctatacgaagttatcgctgaggtctgcctcgtg
YFP11	pCM184 backbone forward	gagctcactagaggatccagccg
YFP12	pCM184 Backbone backward	agatctagacgtcaggtggcacttttc

### Methanol measurements in supernatant

Methanol consumption rates were determined by taking time course samples for supernatant methanol measurements. At least 2 points were taken during exponential phase growth. 750 ul of the supernatant sample were centrifuge filtered through 0.22 μm centrifuge filters (Costar® Spin-X® centrifuge tube filters, cellulose acetate membrane, pore size 0.22 μm, non-sterile, Sigma-Aldrich, (St. Louis, MO) for 2 min at 14,000 rpm. GC-FID (Aglient, Santa Clara, CA) was used for methanol detection with a 6890 Gas Chromatograph equipped with flame ionization detector (FID). Data were collected and converted into matlab input files with LabVIEW 2010. Data analysis was later done in Matlab. SLB-IL60 column (Supelco, Bellefonte, PA, USA) with a 30 m × 0.25 mm inner diameter (i.d.) × 0.2 μm thickness was installed for gas chromatograms. 1 μl of supernatant was injected and separated in the column and then entering into FID detector with a split ratio 50:1. Oven temperatures were programmed as follows. An initial temperature of 120 °C was held for 1 min, and then ramped at 35 °C /min until a final temperature of 200 °C was achieved. The FID was operated at 220 °C. All measurements were done in triplicate. With each run, a new calibration curve was then generated using the same instrument setting.

### Metabolite extraction and biomass hydrolysis for isotopomer distribution measurements

Both proteinogenic amino acids and intracellular metabolites were used for isotopomer measurements in order to obtain good coverage of central carbon metabolism under methylotrophic growth. 20 ml of cell culture were collected and quenched using fast filtration using 0.2 μm nylon membrane filters (0.2 μm, 47 mm from PALL life science, Port Washington, NY) and liquid nitrogen. Cell pellets on 0.22 μm filters were lyophilized using FreeZone 4.5 l benchtop freezedry system (Labconco, Kansas) for 8–12 h to remove medium residue. The filter was incubated in 20 ml of boiling water for 10 min, and then placed on ice for at least 20 min for protein precipitation. The whole broth was then centrifuged at 4 °C, 5000 rpm for 10 min. The clear supernatant was transferred into a clean 50 ml falcon tube, and then frozen using liquid nitrogen. The remaining tubes containing protein and cell debris were centrifuged again, at 4 °C, 5000 rpm for 20 min, and supernatants were removed carefully. Frozen samples were then lyophilized twice, and reconstituted in 100 μl nanopure water. The 100 μl of sample was distributed into 2 vials. One 50 μl sample was used for LC/MS/MS measurements, and the other was dried using a Speedvac (Labconco, Kansas), and derivatized with TBDMS and methoxyamine hydrochloride O-Methylhydroxylamin-hydrochlorid (Sigma-Aldrich, St. Louis, MO) using a previously described method [20]. Cell pellets were then hydrolyzed using 1 ml of 6 N HCl at 105 °C for 22–24 h. The remaining HCl was later evaporated with nitrogen flow. Dried samples were reconstituted in 500 μl nanopure water, and ash and cell debris were then filtered out of the sample using centrifuge tube filter (spin-x costar, 0.22 μm, Sigma-Aldrich). 50 μl of the reconstituted sample was then dried and derivatized using TBDMS as noted above.

Both TBDMS-derivatized proteinogenic amino acids and intracellular metabolites were detected using an Agilent (Santa Clara, CA) GCMS with an Agilent J&W HP-5 ms Ultra Inert GC Column (30 m × 0.25 mm × 0.25 μm, catalog number: 19091 s-433). Each sample was run in duplicate on the GCMS with the same temperature program, and also as one more run with a slower oven temperature ramping rate. The oven temperature was programmed as follows: for regular runs, the oven temperature was programmed as follows:, initial temperature at 100 °C, hold for 4 min, then ramp to 300 °C with ramp rate at 5 °C/min, then hold for 5 min, with total run time 49 min. In order to further separate compounds with similar retention times a slower run was developed. For the slower run, the ramp rate slows down to 2.5 °C/min with total run 89 min. MS source temperature was set at 230 °C and MS quad temperature set as150 °C.

Sugar phosphate was separated by a Zic-pHilic column (SeQuant, PEEK 150 mm length × 2.1 mm metal free, with 5 μm polymeric film thickness) using LC-MS/MS (Xevo, Waters, Milford, MA). Mobile phase A is LC grade water with 2 mM formic acid and 4 mM ammonium, mobile phase B is acetonitrile with 0.1 % formic acid. Column temperature was set at 30 °C. The LC condition starts with 0.15 ml/min flow rate with initial gradient A = 15 %, hold for 2 min, then increased to 40 % over the next 2 min. At 7 min, A = 50 %, at 10 min, A = 80 %, at 11 min, A is set to 90 %, held for 0.5 min, and then switched to 15 % at 11.50 min to re-equilibrate the column for another 4.5 min.

Waters Xevo G2-S Q-Tof equipped with Waters Acquity UPLC I class (Milford, MA) was used for some of the proteinogenic amino acids using HILIC-BEH-amide (Waters Acquity, part number 186004802, with dimension 2.1 mm × 150 mm, 1.7um). Mobile phase A is water with 20 mM ammonium formate and 0.1 % formic acid, mobile phase B is acetonitrile with 0.1 % formic acid. LC solvent gradient was set as follow, starting at 10 % A for 1 min, at 2 min, A = 35 %, at 3 min, A = 40 %, at 6 min, A = 50 %, at 7.5 min, A = 80 %, holding for another 2 min. Then the column is re-equilibrated at 9.51 min by running 10 % A through the column until 5.5 min.

### Central carbon metabolism model construction and ^13^C flux analysis

The central carbon metabolism pathway model used for ^13^C flux analysis is shown in Fig. 1. It was constructed based on a previous model [14], and details are shown in Additional files 2, 3, 4 and 5: Table S2, S3, S4 and S5. The pathways included are: oxidation of methanol to CO_2_ via formaldehyde and formate, conversion of formate to methylene-H_4_F using the H_4_F pathway; assimilation of methylene H_4_F via the serine cycle; the TCA cycle; the ethylmalonyl-CoA pathway; gluconeogenesis; and anaplerotic reactions [reactions catalyzed by pyruvate kinase, PEP carboxylase and malic enzyme] overlapping with the serine cycle in key intermediate metabolites nodes (such as 2-O-Phosphono-D-glyceric acid (2PGA) and phosphoenolpyruvate (PEP). Some of the C3 and C4 metabolites were involved in more than one pathway in this interconnected set of cycles. For instance, 1) 2PGA is the branch node for the serine cycle and gluconeogenesis, 2) PEP and pyruvate are both in the serine cycle and anaplerotic pathways, 3) Malate and OAA were both in the serine cycle, TCA cycle and anaplerotic pathway. This complexity and redundancy offers the cell alternatives for the connections between the serine cycle and EMC pathway under environmental perturbation and gene manipulation.

Methanol uptake rates and biomass yields were measured for individual conditions. Extracellular metabolite secretion was measured using 1H NMR with a previously described protocol [26]. For *in silico* simulation, Influx_s was used [27].

## Abbreviations

AM1, *Methylobacterium extorquens* AM1; HY, hypho medium with 1.35 μM cobalt; HYC, Hypho medium with 6.31 μM

## Additional files

Additional file 1: Table S1. Genomic differences between different *M. extorquens* AM1 strains. Genome of the Marx laboratory strain has been published [22]. (DOCX 17 kb) Additional file 2: Central carbon metabolism model for *M. extorquens* AM1 and list of metabolites with full name. (XLSX 45 kb) Additional file 3: Three replicates of isotopomer labeling pattern of metabolites used in the simulation. (XLSX 77 kb) Additional file 4: Detailed table for simulated flux with absolute values for all four conditions with 3 replicates each. (XLSX 42 kb) Additional file 5: Detailed table for simulated flux distribution with relative values to specific methanol uptake rate for all four conditions with 3 replicates each. (XLSX 44 kb)
