# Supplementary material for: Difference in C3–C4 metabolism underlies tradeoff between growth rate and biomass yield in Methylobacterium extorquens AM1
Source: BMC Microbiol. 2016 Jul 19;16:156. doi: 10.1186/s12866-016-0778-4 (PMC4949768; doi:10.1186/s12866-016-0778-4)
Supplement: Additional file 1: Table S1. — Genomic differences between different M. extorquens AM1 strains. Genome of the Marx laboratory strain has been published [22]. (DOCX 17 kb) [file 12866_2016_778_MOESM1_ESM.docx]

Table S.1 Genomic differences between different *M. extorquens* AM1 strains. Genome of the Marx laboratory strain has been published [22]

| Gene | Description | Position | Change | Chris Marx Modern | LL | VL |
| --- | --- | --- | --- | --- | --- | --- |
| META1_2197 | Methionine aminopeptidase | 2,268,726 | T🡪A |  | X |  |
| META1_2197 | Methionine aminopeptidase | 2,268,739 | +A |  | X |  |
| META1_3425/26  (intergenic) | Unknown//unknown | 3,562,265 | Δ194bp |  | X |  |
| META2_1014 | Putative thiol:disulfide interchange protein | 946,695 | T🡪C |  | X |  |
| META1_4621/20 (intergenic) | Putative SecD/SecF family translocase//*yajC* | 4,746,796 | A🡪G |  |  | X |
| META1_2676 | unknown | 2,803,789 | C🡪T | X |  |  |
| META1_2676 | unknown | 2,803,840 | T🡪C | X |  |  |
| IS elements |  |  |  |  |  |  |
| META1_4587 | ISMex4 insertion into gene of unknown function | 4703837 | insertion |  | X |  |
| META2_1244 | ISMex4 insertion into gene of unknown function | 1155582 | insertion |  | X |  |
| META1_tRNA19 | Ser tRNA | 4056718 | insertion |  |  | X |
| META1_4026/7  (intergenic) | Ser/Thr phosphatase  //unknown | 4115508 | insertion |  |  | X |
